# A series of N-terminal epitope tagged *Hdh* knock-in alleles expressing normal and mutant huntingtin: their application to understanding the effect of increasing the length of normal huntingtin’s polyglutamine stretch on CAG140 mouse model pathogenesis

**DOI:** 10.1186/1756-6606-5-28

**Published:** 2012-08-14

**Authors:** Shuqiu Zheng, Nima Ghitani, Jessica S Blackburn, Jeh-Ping Liu, Scott O Zeitlin

**Affiliations:** 1 Department of Neuroscience, University of Virginia School of Medicine, Charlottesville, VA, 22908, Box 801392, USA; 2 Neuroscience Training Program, University of Wisconsin, Madison, WI, USA; 3 Molecular Pathology, Massachusetts General Hospital/Harvard Medical School, Charlestown, MA, USA

**Keywords:** Huntingtin, Epitope tag, Knock-in, Polyglutamine, Proline-rich region, Sequestration, Huntington’s disease

## Abstract

**Background:**

Huntington’s disease (HD) is an autosomal dominant neurodegenerative disease that is caused by the expansion of a polyglutamine (polyQ) stretch within Huntingtin (htt), the protein product of the HD gene. Although studies in vitro have suggested that the mutant htt can act in a potentially dominant negative fashion by sequestering wild-type htt into insoluble protein aggregates, the role of the length of the normal htt polyQ stretch, and the adjacent proline-rich region (PRR) in modulating HD mouse model pathogenesis is currently unknown.

**Results:**

We describe the generation and characterization of a series of knock-in HD mouse models that express versions of the mouse HD gene (*Hdh*) encoding N-terminal hemaglutinin (HA) or 3xFlag epitope tagged full-length htt with different polyQ lengths (HA7Q-, 3xFlag7Q-, 3xFlag20Q-, and 3xFlag140Q-htt) and substitution of the adjacent mouse PRR with the human PRR (3xFlag20Q- and 3xFlag140Q-htt). Using co-immunoprecipitation and immunohistochemistry analyses, we detect no significant interaction between soluble full-length normal 7Q- htt and mutant (140Q) htt, but we do observe N-terminal fragments of epitope-tagged normal htt in mutant htt aggregates. When the sequences encoding normal mouse htt’s polyQ stretch and PRR are replaced with non-pathogenic human sequence in mice also expressing 140Q-htt, aggregation foci within the striatum, and the mean size of htt inclusions are increased, along with an increase in striatal lipofuscin and gliosis.

**Conclusion:**

In mice, soluble full-length normal and mutant htt are predominantly monomeric. In heterozygous knock-in HD mouse models, substituting the normal mouse polyQ and PRR with normal human sequence can exacerbate some neuropathological phenotypes.

## Background

Huntington’s disease (HD) is an autosomal dominant neurodegenerative disorder that is caused by the expansion of a CAG triplet repeat encoding polyQ (>36Q) within the first exon of the *HTT* gene [1,2]. The length of the expanded polyQ stretch correlates inversely with age at onset, and moderate polyQ expansions (40-50Q) in htt are usually associated with disease onset at middle age. Unfortunately, despite a number of therapies targeted at individual symptoms, there is currently no way to delay or halt progression of the disease and death results ~10-20 years after diagnosis. Neurons in the striatum and deeper layers of the cortex are affected predominantly, although neuronal cell death and white matter loss are also detected in many other areas of the brain [3]. A neuropathological hallmark of HD is the appearance of nuclear and cytoplasmic (neuropil) inclusions of aggregated N-terminal fragments of mutant htt [4-6]. Despite a correlation between the appearance of htt aggregates and behavioral deficits in the majority of HD mouse models, the role of these inclusions in the mechanism of HD pathogenesis is still uncertain, as the results from in vitro experiments and some HD mouse models have suggested that large visible mutant htt inclusions are neuroprotective [7-11]. However, such aggregates not only have the ability to recruit toxic soluble fragments or oligomers of mutant Htt, but they can also sequester other polyQ-containing proteins, including wild-type htt [12-15]. In vitro studies, for example, have demonstrated that aggregates containing both mutant and wild-type htt N-terminal fragments are formed when mutant and wild-type truncated htt expression constructs are co-expressed. A Q_20_ polypeptide can augment Q_47_ aggregation in vitro by enhancing nucleation kinetics, and co-expression of a Q_20_ version of htt exon 1 with Q_93_-htt exon 1 accelerated aggregation and increased toxicity in a *Drosophila* model [16]. Wild-type htt is an essential protein during early embryogenesis, neurogenesis, and in adult neuronal homeostasis. Loss of murine huntingtin (htt) expression results in progressive neurodegeneration [17], increased apoptosis [18], axonal transport deficits in neurons [19,20], altered mitotic spindle orientation in dividing neuronal progenitor cells [21], and hypomorphic primary cilia [22]. Thus, potential sequestration of wild-type htt by mutant htt could contribute to HD pathogenesis via a dominant negative loss-of-function mechanism. Indeed, depletion of wild-type htt in YAC transgenic HD model mice exacerbates deficits in motor function, survival, and striatal neuronal size [23,24]. However, despite evidence from in vitro experiments and a *Drosophila* model supporting the hypothesis that truncated N-terminal fragments of mutant Htt can sequester wild-type htt fragments in aggregates, evidence for the sequestration of wild-type htt by mutant htt in mouse models is lacking.

Immediately adjacent to the htt polyQ stretch is a proline-rich region (PRR) that is thought to have co-evolved in vertebrates with the polyQ stretch [25]. Data from in vitro and cell culture experiments suggest that aggregation of mutant htt N-terminal fragments and potentially sequestration of wild-type htt can also be modulated by the adjacent PRR [26-30]. A normal htt exon 1 construct containing the PRR can ameliorate the toxic effects of an N-terminal 103Q construct, while a construct expressing normal htt exon 1 without the PRR does not [31]. The mouse htt PRR is a 32 amino acid domain consisting of P_3_, P_10_, P_2_, and P_7_ stretches interrupted by short Q-rich stretches 1–3 amino acids in length. The human PRR is slightly longer (38 amino acids) and consists of P_11_ and P_10_ sequences interrupted by a proline-rich 17 amino acid stretch. It is not yet known if expression of a humanized version of normal htt with a non-pathogenic polyQ stretch and the human PRR can influence HD mouse model phenotypes.

To determine the extent of potential dominant-negative interactions in vivo, and to begin to explore the effect of expressing a non-pathogenic humanized allele of *Hdh* encoding htt with a 20Q stretch and human PRR on HD mouse model pathogenesis, we have generated a series of knock-in HD mouse models expressing (1) the mouse HD gene (*Hdh*) encoding full-length normal htt (7Q and mouse PRR) with hemaglutinin (HA) or triple Flag N-terminal epitope tags (HA7Q- and 3xFlag7Q-htt), (2) a humanized normal *Hdh* allele encoding a 3xFlag-tagged version of htt with a 20Q stretch adjacent to the human PRR (3xFlag20Q-htt), and (3) a humanized *Hdh* allele encoding a 3xFlag-tagged version of mutant htt with a 140Q stretch adjacent to the human PRR (3xFlag140Q-htt). By co-immunoprecipitation, we find that soluble full-length murine 7Q-htt does not associate stably with itself or with 140Q-htt. However, we can detect a very low level of interaction between full-length 3xFlag20Q-htt and 140Q-htt. In addition, we observe a significant increase in the number of nuclear inclusions, and in the mean size of aggregates detected in *Hdh*^*140Q/3xFlag20Q*^ mice compared with *Hdh*^*140Q/3xFlag7Q*^ mice. These observations, together with an increase in gliosis and lipofuscin accumulation in the *Hdh*^*140Q/3xFlag20Q*^ brain in comparison to the *Hdh*^*140Q/3xFlag7Q*^ brain, suggest that replacing the mouse polyQ and PRR stretches with normal human sequence can exacerbate some aspects of the CAG140 HD mouse model phenotype.

## Results

**Figure 1:**
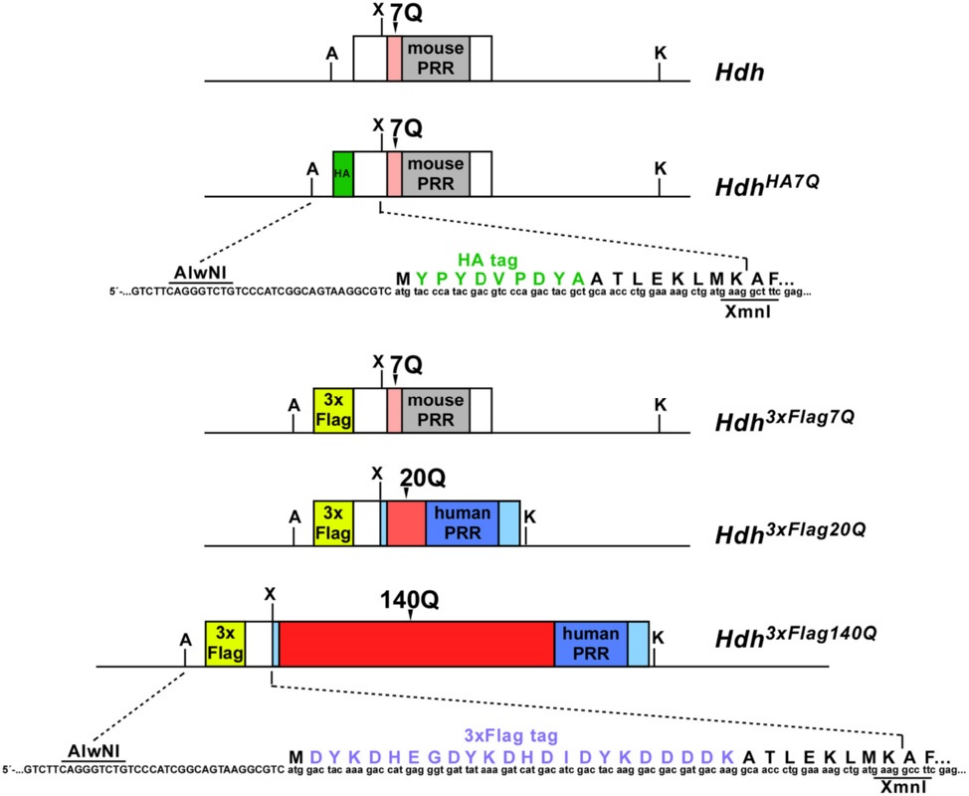
**Diagram of the epitope tag-modified* Hdh *exon 1.** The wild-type * Hdh * exon 1 contains a short polyQ stretch (7Q) and an adjacent proline-rich region (mouse PRR, gray). An HA N-terminal epitope tag (green) was inserted between amino acids 1 and 2 of the * Hdh * exon 1 containing the wild-type mouse polyQ stretch (* Hdh *^* HA7Q *^), while a 3xFlag N-terminal epitope tag (yellow) was inserted in the wild-type * Hdh * exon 1 (* Hdh *^* 3xFlag7Q *^), a chimeric mouse/human exon 1 containing a normal human polyQ stretch (* Hdh *^* 3xFlag20Q *^), and a chimeric mouse/human exon 1 with an expanded polyQ stretch (*Hdh*^* 3xFlag140Q *^). The human portion of the chimeric exon 1 is indicated in light blue and darker blue (the human PRR), while the polyQ stretches are displayed in shades of red. The sequences of the epitope tags are indicated along with key restriction sites used in the construction of the gene targeting vectors. A, * AlwN *I; X, * Xmn *I; K, * Kpn. *I.

To insert N-terminal HA and 3xFlag epitope tags into the *Hdh* locus, we assembled gene targeting constructs by replacing an endogenous *Hdh* exon 1 *AlwN*I – *Xmn*I restriction fragment with a PCR-generated synthetic fragment containing either the HA or 3xFlag epitope tag inserted between htt amino acids 1 and 2. The 140Q stretch was derived from our CAG140 targeting construct [32], and contains the human proline-rich region (PRR) that is adjacent to the polyQ stretch. The 20Q stretch was obtained by PCR amplification of a human wild-type allele using the same procedure employed to isolate the CAG140 stretch, and it also contains a human PRR (Figure 1). Germline transmission was obtained from three independent ES cell clones (*Hdh*^*HA7Q*/+^and *Hdh*^*3xFlag7Q/+*^) or two independent clones (*Hdh*^*3xFlag20Q/+*^ and *Hdh*^*3xFlag140Q/+*^). All mice have been backcrossed to the C57BL/6J strain for at least six generations. 

To establish that the addition of the HA or 3xFlag N-terminal epitope tags did not affect htt’s essential functions during embryonic development, the genotypes of progeny from heterozygous intercrosses were evaluated for any deviation from the expected Mendelian frequency (Table 1). Homozygous *Hdh*^*HA7Q/HA7Q*^, *Hdh*^*3xFlag7Q/3xFlag7Q*^, and *Hdh*^*3xFlag20Q/3xFlag20Q*^ mice were obtained with the expected frequency, and mice hemizygous for the 3xFlag epitope-tagged 7Q allele (*Hdh*^*3xFlag7Q/-*^) were obtained from crosses with *Hdh*^*+/−*^ mice. *Hdh*^*3xFlag140Q/3xFlag140Q*^ progeny were also obtained from heterozygous intercrosses, but the number of homozygous progeny trended less than that predicted by the Mendelian frequency, (this difference, however, did not reach significance in the χ^2^ test; Table 1), suggesting the possibility for either increased toxicity or that 3xFlag140Q-htt may not function as efficiently as 3xFlag7Q-htt during embryonic and early postnatal development. Adult *Hdh*^*3xFlag7Q/-*^ did not exhibit any obvious behavioral or motor abnormalities, but future testing is needed to determine if subtle behavioral phenotypes exist. Gross morphology of *Hdh*^*HA7Q/HA7Q*^, *Hdh*^*3xFlag7Q/3xFlag7Q*^, *Hdh*^*3xFlag20Q/3xFlag20Q*^, and *Hdh*^*3xFlag140Q/+*^ brains at 6 months of age were normal (data not shown).

**Table 1:** Chi square analyses of genotypes produced from heterozygous and hemizygous crosses

**Genotype**	**# Observed (# Expected)**	**χ**^**2**^**(*P*)**
Cross: *Hdh*^*3xFlag7Q/+*^ X *Hdh*^*3xFlag7Q/+*^		
+/+	16 (23)	1.69 (0.429)
3xFlag7Q/+	51 (46)	
**3xFlag7Q/3xFlag7Q**	**26 (23)**	
Cross: *Hdh*^*HA7Q/+*^ X *Hdh*^*HA7Q/+*^		
+/+	16 (20)	1.01 (0.603)
HA7Q/+	39 (40)	
**HA7Q/HA7Q**	**25 (20)**	
Cross: *Hdh*^*3xFlag20Q/+*^ X *Hdh*^*3xFlag20Q/+*^		
+/+	6 (6)	0.0697 (0.966)
3xFlag20Q/+	12 (12)	
**3xFlag20Q/3xFlag20Q**	**5 (6)**	
Cross: *Hdh*^*3xFlag7Q/+*^ X *Hdh*^*+/-*^		
+/+	2 (4)	1.15 (0.765)
3xFlag7Q/+	6 (4)	
**3xFlag7Q/-**	**4 (4)**	
+/-	5 (4)	
Cross: *Hdh*^*3xFlag140Q/+*^ X *Hdh*^*3xFlag140Q/+*^		
+/+	9 (10)	2.03 (0.362)
3xFlag140Q/+	24 (20)	
**3xFlag140Q/3xFlag140Q**	**5 (10)**	

The observed and expected numbers of the homozygous and hemizygous progeny are in bold.

**Figure 2:**
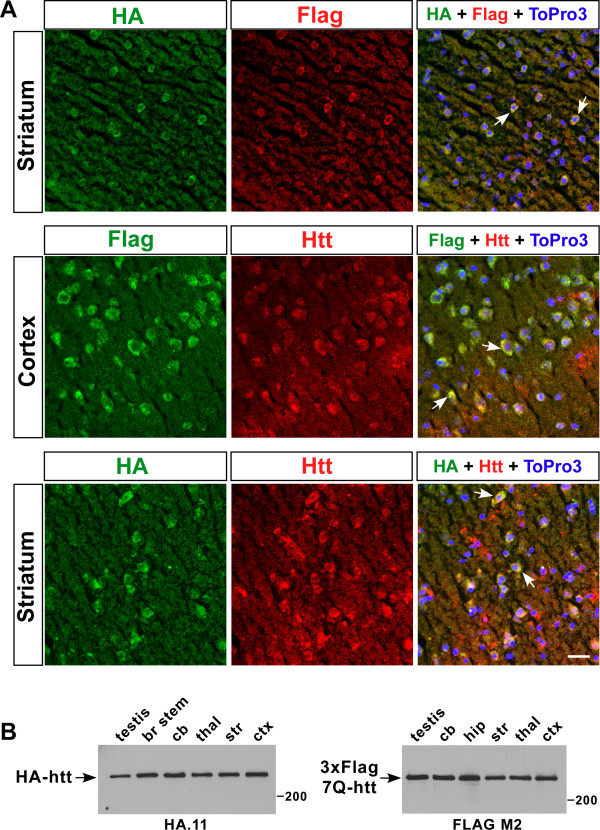
**Expression of HA7Q-htt and 3xFlag7Q-htt in selected brain regions.** (**A**) Confocal microscopy of HA epitope (HA), 3xFlag7Q epitope (FLAG), and htt (Htt) expression in the 24 month-old * Hdh *^* 3xFlag7Q/HA7Q *^ striatum and cortex. Nuclei were visualized with the DNA dye To-Pro-3 (blue). Arrows indicate examples of where co-localization of the HA and 3xFlag epitopes, or of the epitopes with htt occurs. Scale bar = 25 μm. (**B**) Western blot analysis of HA7Q-htt (left panel) and 3xFlag7Q-htt (right panel) in 50 μg total protein isolated from * Hdh *^* HA7Q/+ *^ and * Hdh *^* 3xFlag7Q/+ *^ testis and various brain regions (brain stem: br stem, cerebellum: cb, thalamus: thal, striatum: str, and cortex: ctx). The antibodies used (monoclonal antibodies HA.11 and MAb FLAG M2) and the position of a 200 kD protein marker are indicated.

Confocal analysis of fresh frozen brain sections from *Hdh*^*3xFlag7Q/HA7Q*^ mice at 24 months of age revealed that HA7Q- and 3xFlag7Q-htt expression could be detected using epitope tag-specific antibodies (Figure 2A). As expected, there is extensive overlap in the expression of both epitopes in the *Hdh*^*3xFlag7Q/HA7Q*^ brain. HA and 3xFlag epitopes are detected predominantly in the soma and neuropil, and their expression overlaps with htt expression that is visualized with an anti-htt antibody (MAB2166). Western analyses of HA7Q- and 3xFlag7Q-htt expression in dissected brain regions, and in testis from 1 month old *Hdh*^*HA7Q/+*^ and *Hdh*^*3xFlag7Q/+*^ mice, respectively, revealed that the epitope-tagged versions of htt can be detected using the appropriate anti-epitope tag antibodies (Figure 2B).

**Figure 3:**
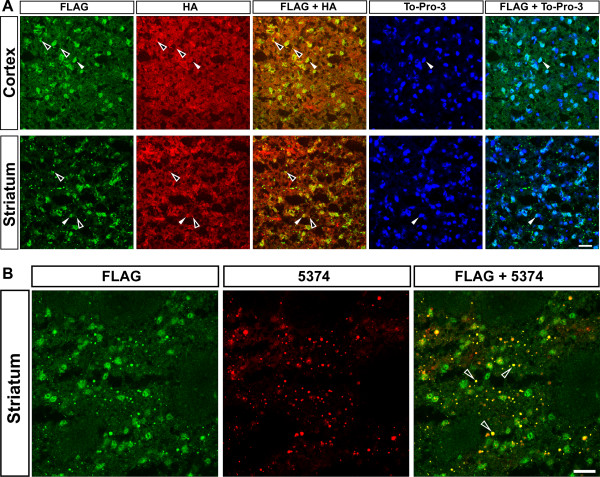
**Expression of 3xFlag140Q-htt and HA7Q-htt in 12 month-old* Hdh ***^*** 3xFlag140Q/HA7Q ***^**cortex and striatum.** (**A**) Confocal microscopy of fresh frozen brain sections containing the striatum and piriform cortex were immunostained with rabbit anti-Flag (green) and mouse anti-HA antibodies (red), while nuclei were visualized with the DNA dye To-Pro-3 (blue). In both the cortex and striatum, the Flag antibody stains a fraction of the nuclei, punctae, and to a lesser extent, the neuropil. HA staining is found diffusely in both the neuropil and in some nuclei. A few punctae co-stain with both Flag and HA antibodies in the cortex and striatum (open arrowheads), and filled arrowheads mark examples of nuclei co-staining with both antibodies. (**B**) In striatal sections co-immunostained with both Flag and an aggregation-specific antibody (mEM48; MAB5374), the majority of htt inclusions in the neuropil are stained with both antibodies (open arrowheads). Scale bar = 25 μm.

To visualize simultaneously both normal (7Q) and mutant (140Q) htt expression using anti-epitope tag antibodies, fresh frozen brain sections from 12 month old *Hdh*^*3xFlag140Q/HA7Q*^ mice were analyzed by confocal microscopy following incubation with FLAG M2 and HA antibodies to detect 3xFlag140Q- and HA7Q-htt, respectively. Htt inclusions were visualized with the aggregation-specific mEM48 (MAB5374) antibody, and nuclei were stained with the fluorescent DNA dye, To-Pro-3 (Figure 3A). In cortex, diffuse and bright punctate neuropil staining , in addition to nuclear staining in a portion of the cells, was observed with the FLAG antibody. In contrast, diffuse HA7Q-htt staining was observed in the neuropil, and in nuclei that also stained with the 3xFlag epitope. HA staining was also detected in several punctae that co-stained with the Flag antibody. Flag staining was present in the neuropil, in a subset of nuclei (Figure 3A), and was detected in neuropil, perinuclear, and nuclear punctae that co-stained with the mEM48 antibody (Figure 3B). Thus, the 3xFlag epitope can be detected in presumptive soluble mutant htt, nuclear inclusions, and neuropil inclusions, while the HA epitope can be detected predominantly in the neuropil, in a subset of nuclei that also stained with mutant htt, and in some htt inclusions.

### Soluble full-length 7Q-htt does not interact with itself or with full-length 140Q-htt

**Figure 4:**
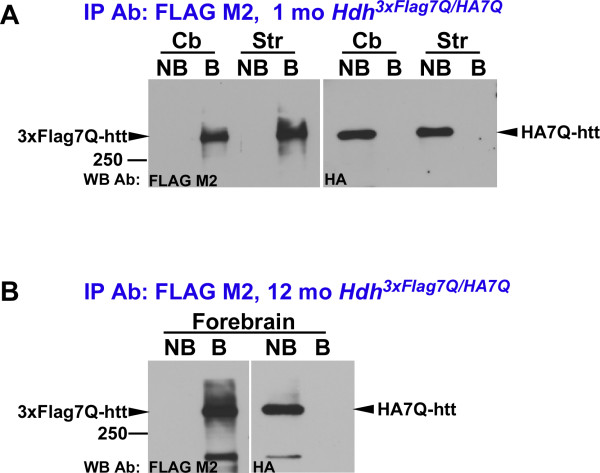
**Full length 3xFlag7Q-htt and HA7Q-htt do not co-immunoprecipitate.** 0.5 mg cytoplasmic extract prepared from the cerebellum (Cb) and striatum (Str) of a 1 month-old * Hdh *^* 3xFlag7Q/HA7Q *^ mouse (**A**) and from the forebrain of a 12 month-old *Hdh*^* 3xFlag7Q/HA7Q *^ mouse (**B**) was immunoprecipitated with anti-FLAG M2 agarose beads. The antibody non-bound sample (NB) represents 10% of the total NB fraction, and the antibody-bound sample (**B**) represents 33% of the total B sample. Protein was resolved on 5% SDS-PAGE, transferred to PVDF membranes, and probed with either FLAG M2 or HA.11 antibodies. In both the 1month- and 12 month-old mice, HA7Q-htt was found entirely in the NB fraction suggesting that there is no stable interaction between full-length soluble 7Q-htt. The positions of htt, and a 250 kD protein size standard are indicated.

Prior in vitro observations, cell culture studies, and experiments in *Drosophila* have demonstrated that mutant htt can sequester normal htt in protein aggregates, but recent analyses of human postmortem brain extracts and protein extracts derived from HD mouse models using blue native polyacrylamide gel eletrophoresis (BNP) have shown that full-length normal and mutant htt are predominantly monomeric [33]. To confirm that epitope-tagged normal htt exists predominantly as a monomer in our mouse models, we immunoprecipitated 3xFlag7Q-htt from cerebellar and striatal protein extracts prepared from 1 month-old *Hdh*^*3xFlag7Q/HA7Q*^ mice, and forebrain extracts prepared from 12 month-old *Hdh*^*3xFlag7Q/HA7Q*^ mice (Figure 4). Flag antibody-bound and non-bound fractions were then analyzed by western blotting using FLAG and HA antibodies. In both 1 month and 12 month-old mice, Flag3x7Q-htt was efficiently immunoprecipitated. HA7Q-htt, in contrast, was detected only the antibody non-bound fractions. Thus, in both young and older mice, epitope-tagged htt exists primarily as a monomer in our protein extracts using our co-immunoprecipitation conditions. 

**Figure 5:**
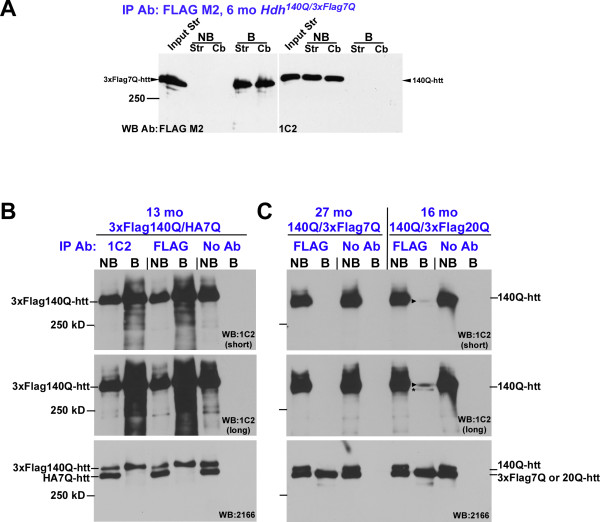
**A low level of interaction between full-length 140Q-htt and 3xFlag20Q-htt but not 3xFlag 7Q-htt can be detected by co-immunoprecipitation.** (**A**) 700 μg of cytoplasmic protein prepared from the striatum (Str) and cerebellum (Cb) of a 6 month-old * Hdh *^* 140Q/3xFlag7Q *^ mouse was immunoprecipitated with anti-Flag agarose beads, and separated into antibody non-bound (NB) and antibody-bound (**B**) fractions. Duplicate samples (Input Str represents 56 μg protein, while each NB sample represents 8% of the fraction, and each B sample represents 10% of the fraction for the anti-FLAG blot, and 40% of the fraction for the 1C2 blot) were fractionated on 5% SDS-PAGE and analyzed by western blotting with the indicated antibodies. 140Q-htt was not detected in the B-fractions, suggesting that a stable interaction between 140Q-htt and 3xFlag7Q-htt was below detection limits. (B) 500 μg whole brain cytoplasmic extract from a 13 month-old * Hdh *^* 3xFlag140Q/HA7Q *^ mouse was immunoprecipitated with anti-FLAG or 1C2 antibodies using Protein G-agarose beads, and the NB- and B-fractions were analyzed by western blotting using 1C2 and 2166 antibodies. (**C**) 500 μg whole brain cytoplasmic extract from 27 month-old * Hdh *^* 140Q/3xFlag7Q *^ and 16 month-old * Hdh *^* 140Q/3xFlag20Q *^ mice was immunoprecipitated with anti-FLAG antibody and Protein-G agarose beads, and the NB- and B-fractions were analyzed by western blotting using 1C2 and 2166 antibodies. Shorter and longer exposures of the western blots are shown in the top two panels of (**B**,**C**). As a negative control for non-specific binding of htt to the agarose beads, antibody was omitted from the mixture of protein extract and Protein G-agarose beads (No Ab) in (**B**,**C**). An arrowhead marks the position of 140Q-htt co-immunoprecipitating with 3xFlag20Q-htt, while an asterisk indicates 3xFlag20Q-htt that is detected inefficiently by the 1C2 antibody when it is present in large amounts (**C**). The position of a 250 kD protein standard is indicated on the left.

We next prepared striatal and cerebellar protein extracts from 6 month-old *Hdh*^*140Q/3xFlag7Q*^ mice to determine if soluble full-length mutant htt can interact with normal htt. Following immunoprecipitation of 3xFlag7Q-htt with anti-FLAG agarose beads, antibody-bound and non-bound fractions were analyzed by western blotting using Flag and expanded polyQ (1C2) antibodies (Figure 5A). While 3xFlag7Q-htt was enriched in the antibody bound fractions, 140Q-htt was detected only in the antibody non-bound fraction. We interpret this result to suggest that little, if any, stable interaction between normal and mutant htt occurs in the 6 month-old *Hdh*^*140Q/3xFlag7Q*^ brain. To determine if an association between normal and mutant htt can be detected in older *Hdh*^*140Q/3xFlag7Q*^ or *Hdh*^*3xFlag140Q/HA7Q*^ mice, co-immunoprecipitation experiments were performed using whole brain extracts prepared from 13 month-old *Hdh*^*3xFlag140Q/HA7Q*^, and 27 month-old *Hdh*^*140Q/3xFlag7Q*^ mice. Cytoplasmic extract from the *Hdh*^*3xFlag140Q/HA7Q*^ and *Hdh*^*140Q/3xFlag7Q*^ brains was immunoprecipitated with anti-expanded polyQ (1C2) or Flag antibodies, using Protein G-agarose beads, and western blots of antibody-bound and non-bound fractions were probed with 1C2 (recognizing 3xFlag140Q-htt and 140Q-htt), and MAB2166 (recognizing both normal and mutant htt) antibodies (Figure 5B, C). HA7Q-htt did not co-immunoprecipitate with 3xFlag140Q-htt, and similarly 140Q-htt did not co-immunoprecipitate with 3xFlag7Q-htt, suggesting that in older mice, a stable interaction between full-length 7Q-htt and 140Q-htt does not occur. To control for non-specific association of htt with the Protein G agarose-beads, whole extract was incubated with Protein G-agarose beads in the absence of 1C2 or Flag antibodies, and the antibody-bound and non-bound fractions were probed with 1C2 and 2166 antibodies (Figure 5B, C). Htt was not detected in the control bound fractions, indicating that little, if any, non-specific binding of htt to the agarose beads occurred.

### A low level of interaction between full-length 20Q-htt and 140Q-htt can be detected by co-immunoprecipitation

To determine if normal htt with a 20Q stretch and human PRR can interact with full-length mutant htt, we performed FLAG immunoprecipitations with Protein-G agarose beads using whole brain extracts prepared from 16 month-old *Hdh*^*140Q/3xFlag20Q*^ mice, and probed the Flag antibody-bound and non-bound fractions with 1C2 and 2166 antibodies (Figure 5C). A very low level of interaction between 140Q- and 3xFlag20Q-htt was detected in the whole brain protein extracts. We estimate that under our co-immunoprecipitation conditions, < 2.5% of soluble full-length mutant htt was associated with 3xFlag20Q-htt (see Methods).

### Sequestration of normal htt in *Hdh*^*140Q/3xFlag7Q*^ and *Hdh*^*140Q/3xFlag20Q*^ striatal inclusions

**Figure 6:**
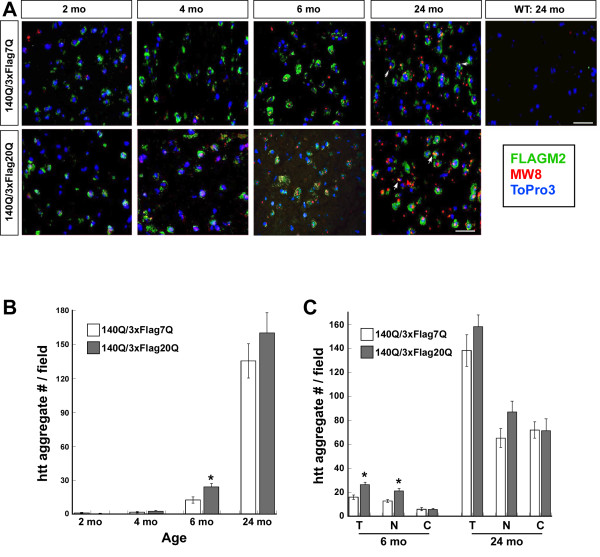
**Time course and quantification of htt inclusions in the* Hdh ***^*** 140Q/3xFlag7Q ***^**and* Hdh ***^*** 140Q/3xFlag20Q ***^**striatum.** (**A**) Representative confocal images of the striatum at 2 months, 4 months, 6 months, and 24 months of age. An image from a 24 month-old wild-type (WT) control is also shown for comparison. 3xFlag7Q- and 3xFlag20Q-htt were detected with the FLAG M2 antibody (green), while htt inclusions were visualized using the MW8 antibody (red). Nuclei were visualized with To-Pro-3 (blue). White arrows indicate inclusions co-staining with the Flag antibody. Scale bar = 25 μm. (**B**) Quantification of total htt aggregate number/imaging field. (**C**) Quantification of total htt aggregate number (T), nuclear aggregate number (N) and neuropil aggregate number per imaging field at 6 months and 24 months of age (n=3 mice for each age and genotype). A significant difference in aggregate number between the * Hdh *^* 140Q/3xFlag7Q *^ and * Hdh *^* 140Q/3xFlag20Q *^ striatum was observed in the nuclear compartment at 6 months of age. Error bars=SEM, * P <0.05.

To explore the effect of expressing a version of htt with a normal human polyQ stretch and human PRR on HD mouse model phenotypes, we first compared the number of striatal neuropil and nuclear inclusions in *Hdh*^*140Q/3xFlag7Q*^ and *Hdh*^*140Q/3xFlag20Q*^ mice at 2 months, 4 months, 6 months, and 24 months of age (n=4 of each genotype) (Figure 6). For both genotypes, htt inclusions were first detected using the MW8 aggregation-specific antibody at 6 months of age, and at this age, the number of nuclear inclusions was greater than that of the neuropil aggregates in both genotypes. By 24 months of age, there was a widespread increase in the number of striatal nuclear and neuropil aggregates in both genotypes (Figure 6). Interestingly, the total number of inclusions in the 6 month-old *Hdh*^*140Q/3xFlag20Q*^ striatum was significantly greater than that found in the *Hdh*^*140Q/3xFlag7Q*^ striatum (Figure 6B). This difference was due entirely to an increase in the number of nuclear inclusions in the *Hdh*^*140Q/3xFlag20Q*^ brain, as there was no significant difference in the number of neuropil aggregates between the two genotypes (Figure 6C).

**Figure 7:**
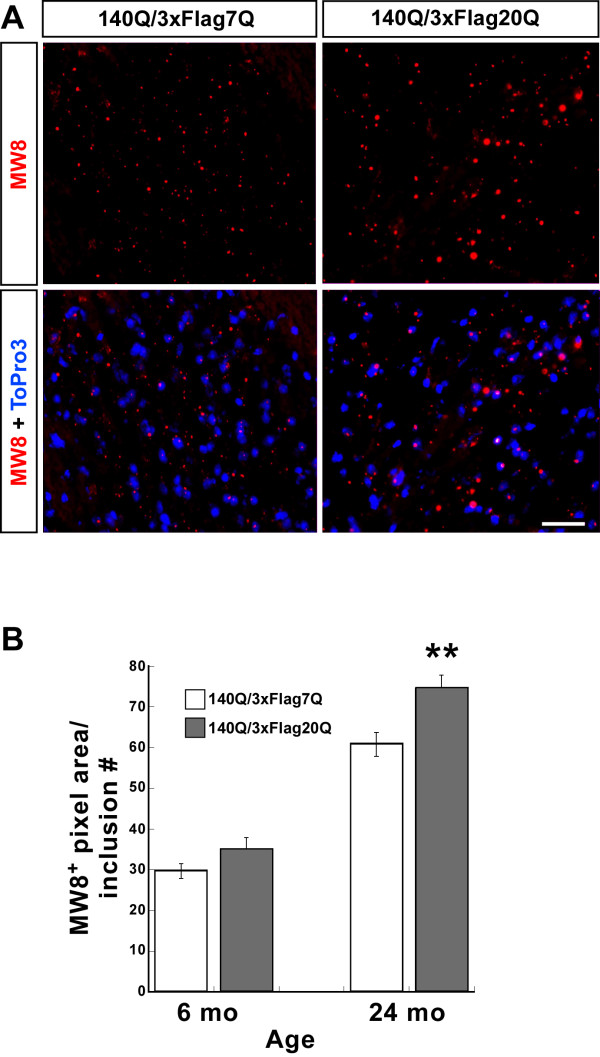
**The mean size of htt inclusions in the* Hdh ***^*** 140Q/3xFlag20Q ***^**striatum is significantly larger than that in the* Hdh ***^*** 140Q/3xFlag7Q ***^**striatum at 2 years of age.** (**A**) Confocal images of the * Hdh *^* 140Q/3xFlag7Q *^ and * Hdh *^* 140Q/3xFlag20Q *^ striatum at 24 months of age. Htt inclusions were detected with the MW8 antibody (red) and nuclei with To-Pro-3 (blue). Scale bar = 25 μm. (**B**) Quantification of the average size (total MW8 ^+^ pixels/inclusion number per imaging field) of htt inclusions in the * Hdh *^* 140Q/3xFlag7Q *^ and * Hdh *^* 140Q/3xFlag20Q *^ striatum at 6 months and 24 months of age (n=3 for each age and genotype, mean ± SEM plotted). ** P <0.01.

Although the number of nuclear and neuropil aggregates was not significantly different in the 24 month-old *Hdh*^*140Q/3xFlag7Q*^ and *Hdh*^*140Q/3xFlag20Q*^ brains, the size of the inclusions appeared larger in the *Hdh*^*140Q/3xFlag20Q*^ striatum (Figure 6A, 7A). To quantify the size of the aggregates, we determined the average MW8^+^ pixel area per aggregate in confocal images by dividing the total MW8^+^ pixel area by the number of aggregates (Figure 7B). At 6 months of age, no significant difference in the mean size of aggregates was observed in the *Hdh*^*140Q/3xFlag7Q*^ and *Hdh*^*140Q/3xFlag20Q*^ striatum. At 24 months of age, however, we observed a significant increase in the mean aggregate size in the *Hdh*^*140Q/3xFlag20Q*^ striatum in comparison to the *Hdh*^*140Q/3xFlag7Q*^ striatum.

**Figure 8:**
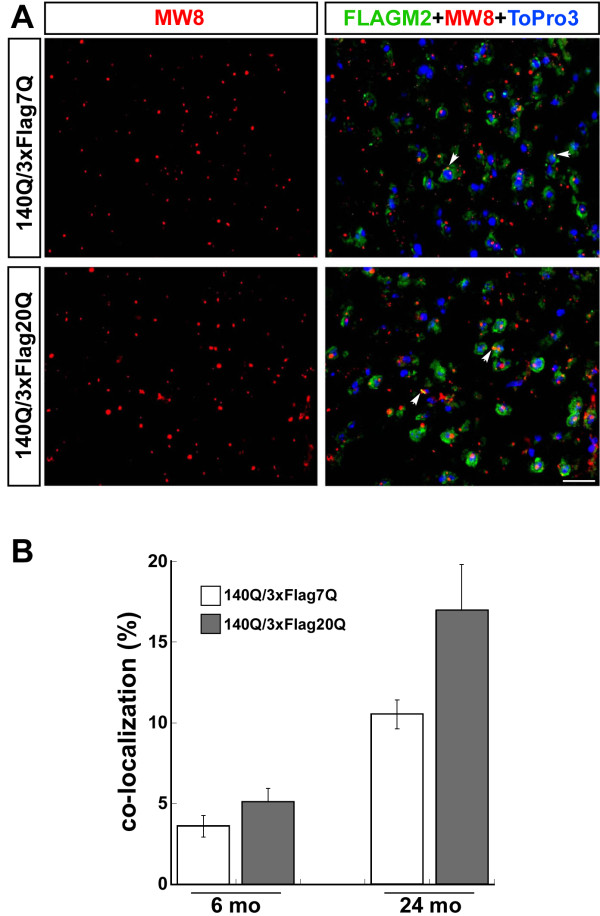
**Co-localization of 3xFlag7Q- and 3xFlag20Q-htt with 140Q-htt in htt inclusions.** (**A**) Confocal images of the * Hdh *^* 140Q/3xFlag7Q *^ and * Hdh *^* 140Q/3xFlag20Q *^ striatum at 24 months of age. Htt inclusions were detected with MW8 (red), while a Flag antibody was used to detect htt with the normal polyQ stretch. White arrows indicate examples of inclusions that contain the 3xFlag epitope (yellow). Scale bar = 25 μm. (**B**) The % co-localization between the Flag epitope and htt inclusions (Flag^+^ MW8^+^ inclusions/MW8^+^ inclusions per microscopic field) (n=4 mice of each genotype, mean ± SEM plotted ). Although there appeared to be a trend for slightly increased co-localization of 3xFlag20Q-htt with the MW8^+^ inclusions at 24 months of age, a significant difference between the two genotypes was not found (* P *=0.06).

To compare the extent of the co-localization of 3xFlag20Q-htt with 140Q-htt vs. 3xFlag7Q-htt with 140Q-htt in aggregates, we quantified the percentage of htt aggregates that co-stained with both Flag and MW8 antibodies in the *Hdh*^*140Q/3xFlag7Q*^ and *Hdh*^*140Q/3xFlag20Q*^ striatum using confocal microscopy (Figure 8A). At 6 months of age, we observed no significant difference in the percentage of htt aggregates that contained the 3xFLAG epitope in the *Hdh*^*140Q/3xFlag7Q*^ and *Hdh*^*140Q/3xFlag20Q*^ striatum (Figure 8B). At 24 months of age, there was a trend towards increased co-localization of epitope-tagged normal htt with mutant htt in the *Hdh*^*140Q/3xFlag20Q*^ brain in comparison to the *Hdh*^*140Q/3xFlag7Q*^ brain (*P*=0.06) (Figure 8B). This result suggests that 3xFlag20Q-htt may be recruited into inclusions more efficiently than 3xFlag7Q-htt, and this could contribute to the increased average size of aggregates we observed in the *Hdh*^*140Q/3xFlag20Q*^ striatum.

**Figure 9:**
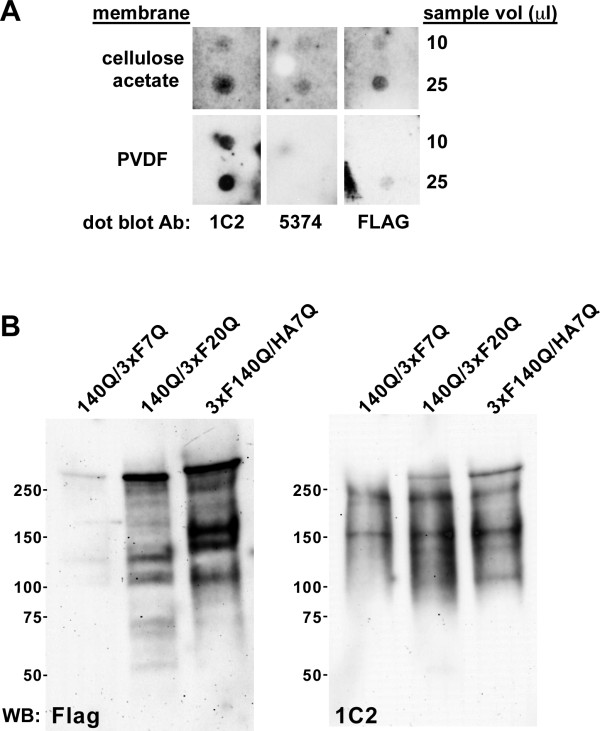
**Detection of 3xFlag7Q- and 3xFlag20Q-htt in the SDS-insoluble nuclear fractions from* Hdh ***^*** 140Q/3xFlag7Q ***^**and* Hdh ***^*** 140Q/3xFlag20Q ***^**brains.** (**A**) Aliquots of the crude nuclear fraction obtained from the whole brain of a 24 month-old * Hdh *^* 140Q/3xFlag7Q *^ mouse were heated in the presence of SDS and analyzed by cellulose filter trap assay and western blotting. SDS-resistant htt inclusions trapped on the cellulose acetate membrane were detected with 1C2 and MAB5374 antibodies, while sequestered 3xFlag7Q-htt was detected with the FLAG M2 antibody. SDS-soluble 140Q-htt and 3xFlag7Q-htt species were detected on the PVDF membrane using 1C2 and FLAG antibodies. (**B**) Crude nuclear fractions were obtained 24 month-old * Hdh *^* 140Q/3xFlag7Q *^ (140Q/3xF7Q), *Hdh*^* 140Q/3xFlag20Q *^ (140Q/3xF20Q), and * Hdh *^* 3xFlag140Q/HA7Q *^ (3xF140Q/HA7Q) brains, boiled in the presence of SDS, sonicated, and the remaining SDS-insoluble material was extracted with formic acid. The formic acid-solubilized fractions were analyzed by western blotting using Flag (FLAG M2, left panel), and 1C2 antibodies (right panel). 3xFlag epitope immunoreactivity in the *Hdh*^* 140Q/3xFlag20Q *^ nuclear fraction is increased in comparison to the * Hdh *^* 140Q/3xFlag7Q *^ fraction. The sizes of protein standards (in kD) are indicated.

To confirm that normal htt was sequestered in mutant htt inclusions, we first performed cellulose acetate filter trap assays [34] with the initial 800xg pellet fraction from 24 month-old *Hdh*^*140Q/3xFlag7Q*^ whole brain extracts (containing crude nuclei and insoluble htt aggregates) (Figure 9A). 1C2, mEM48, and Flag-positive htt inclusions trapped on the cellulose acetate were detected by western blotting, while an underlying PVDF membrane was positive for SDS-soluble 140Q- and 3xFlag7Q-htt species, but negative for mEM48-positive aggregates. 

In both full-length and truncated mutant htt HD mouse models, the predominant species of mutant htt found in macroscopic inclusions consists of N-terminal fragments [35-37]. To determine if epitope-tagged N-terminal fragments of normal htt were present in the inclusions, we extracted the SDS-insoluble 800xg pellet fraction obtained from 24 month old *Hdh*^*140Q/3xFlag7Q*^, *Hdh*^*140Q/3xFlag20Q*^, and *Hdh*^*3xFlag140Q/HA7Q*^ brains with formic acid to solubilize the htt aggregates [38-40], and performed western blotting using Flag and 1C2 antibodies to visualize full-length and N-terminal fragments of normal and mutant htt (Figure 9B). In the formic acid-solubilized SDS-insoluble nuclear fractions, we could detect 140Q- and 3xFlag140Q-htt fragments with the 1C2 antibody that ranged in size from ~100 kD to ~250 kD, and apparently full-length mutant htt. The Flag antibody detected an increased level of 3xFlag20Q-htt fragments in comparison to 3xFlag7Q-htt fragments. We interpret these data to suggest that sequestration of normal htt N-terminal fragments into inclusions does occur in heterozygous knock-in HD mouse models, and is enhanced by expression of a humanized version of the normal *Hdh* allele.

### Increased formation of small neuropil htt aggregates occurs when mouse htt’s 7Q stretch and PRR are replaced by a 20Q stretch and the human PRR in *Hdh*^*140Q/3xFlag20Q*^ mice

**Figure 10:**
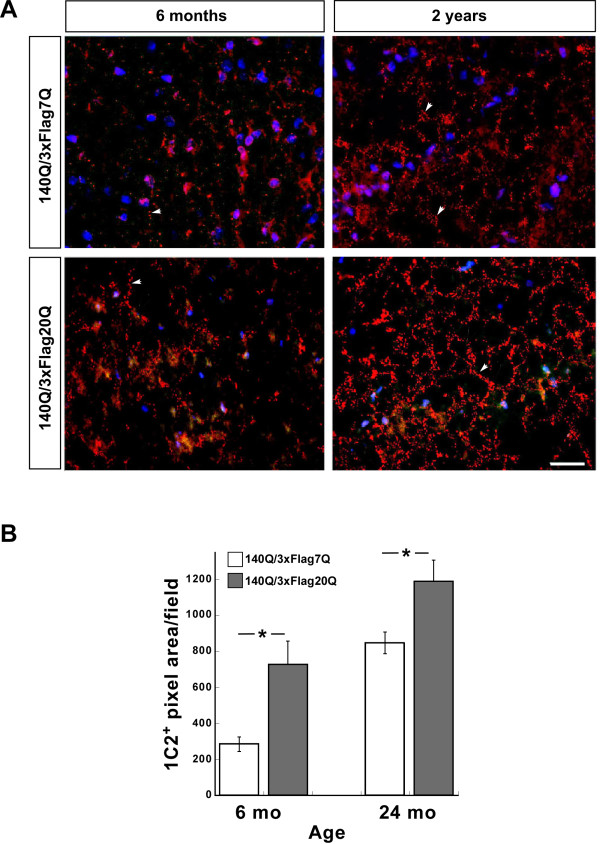
**Formic acid treatment reveals an increase in small 1C2**^**+**^**inclusions in the* Hdh ***^*** 140Q/3xFlag20Q ***^**striatum in comparison to* Hdh ***^*** 140Q/3xFlag7Q ***^**striatum.** (**A**) Images of the striatum from 6 month-old and 2 year-old * Hdh *^* 140Q/3xFlag7Q *^ and * Hdh *^* 140Q/3xFlag20Q *^ mice probed with 1C2 antibody (red) following formic acid treatment. Nuclei were visualized with To-Pro-3. Examples of “aggregate chains” are indicated with white arrows. Scale bar = 25 μm. (**B**) Quantification of the total 1C2^+^ pixel area per microscopic field in the striatum of * Hdh *^* 140Q/3xFlag7Q *^ and * Hdh *^* 140Q/3xFlag20Q *^ mice at 6 months and 24 months of age (n=3 mice for each age and genotype, mean ± SEM). * P <0.05.

The large neuropil and nuclear inclusions that we visualized by standard immunohistochemical methods using the MW8 and mEM48 (MAB5374) antibodies may not represent all the sites where aggregates are forming. To visualize these sites, treatment of tissue sections with formic acid to expose the expanded polyQ epitope within the htt aggregates prior to antibody staining is required [41]. Using this method, small neuropil aggregates and aggregation foci located in striatal neuronal processes resembling chains of immuno-positive punctae were observed [41]. Such 1C2^+^ punctae were detected in both *Hdh*^*140Q/3xFlag7Q*^ and *Hdh*^*140Q/3xFlag20Q*^ striata (Figure 10A). For quantification, we measured the total pixel area of the 1C2^+^ punctae, as they were difficult to resolve clearly. We observed a significant increase in 1C2 staining following formic acid treatment in the *Hdh*^*140Q/3xFlag20Q*^ striatum in comparison to the *Hdh*^*140Q/3xFlag7Q*^ striatum at both 6 months and 24 months of age (Figure 10B). Unfortunately, we could not detect co-localization of the 3xFlag epitope with the expanded polyQ epitope, as we were unable to recover FLAG immunoreactivity following formic acid treatment (data not shown). Nevertheless, our observations suggest that increasing the length of the normal htt polyQ stretch and/or replacing the mouse PRR with the human PRR can enhance the formation of small cytoplasmic htt aggregates. 

### Increased astrocytosis and lipofuscin accumulation is observed in *Hdh*^*140Q/3xFlag20Q*^ mice compared to *Hdh*^*140Q/3xFlag7Q*^ mice

**Figure 11:**
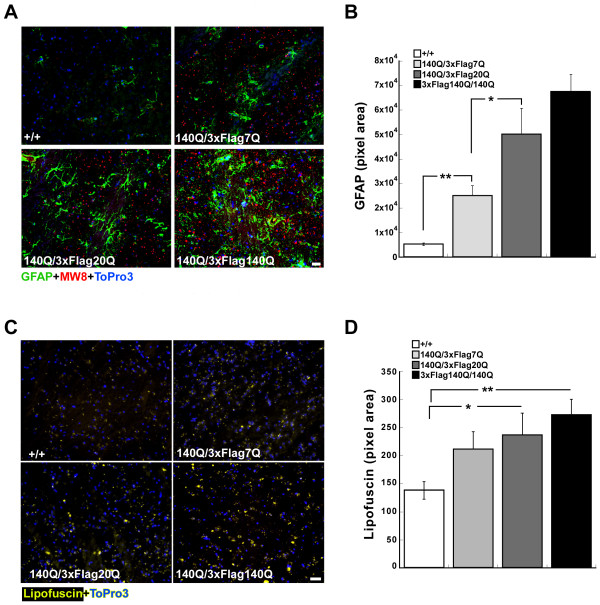
**Gliosis and lipofuscin accumulation in* Hdh ***^*** 140Q/3xFlag20Q ***^**and* Hdh ***^*** 140Q/3xFlag7Q ***^**mice.** (**A**) Images of the striatum of 2 year-old wild type (+/+), * Hdh *^* 140Q/3xFlag7Q *^, *Hdh*^* 140Q/3xFlag20Q *^, and * Hdh *^* 140Q/3xFlag140Q *^ mice probed with antibodies against GFAP (green) and htt inclusions (MW8, red). Nuclei were visualized with To-Pro-3 (blue). (**B**) The mean total pixel area of GFAP staining per microscopic field of the ventral striatum was plotted for each genotype (n=3 mice for each genotype). (**C**) Images of lipofuscin autofluorescence (yellow) in the striatum of 2 year-old wild type (+/+), * Hdh *^* 140Q/3xFlag7Q *^, * Hdh *^* 140Q/3xFlag20Q *^, and * Hdh *^* 140Q/3xFlag140Q *^ mice. Nuclei were stained with To-Pro-3. (**D**) The mean total pixel area of lipofuscin autofluorescence was plotted for each genotype (n=3 mice for each genotype, mean ± SEM plotted). *P<0.05, **P<0.01 in **B,D**. Scale bar = 25 μm in **A,C**.

In HD mouse models and in the HD brain, reactive astrocytosis occurs as a consequence of mutant htt expression [42-44]. To determine if astrocytosis in *Hdh*^*140Q/3xFlag20Q*^ mice is altered in comparison to that observed in *Hdh*^*140Q/3xFlag7Q*^ mice, we examined glial fibrillary acidic protein (GFAP) expression by immunohistochemistry in the striatum of 24 month-old wild-type, *Hdh*^*140Q/3xFlag7Q*^, *Hdh*^*140Q/3xFlag20Q*^, and *Hdh*^*140Q/3xFlag140Q*^ mice (Figure 11A). As expected, GFAP immunostaining was relatively low in the wild type striatum (measured as total pixel area of GFAP signal/field), likely reflecting the basal expression of GFAP in resident astrocytes. In all three HD mouse models, however, GFAP immunostaining was significantly higher in the mutant striatum in comparison to the wild type striatum (Figure 11B). A significant increase in astrocytosis was observed in the *Hdh*^*140Q/3xFlag7Q*^ striatum compared to wild-type striatum, and in the *Hdh*^*140Q/3xFlag20Q*^ striatum compared to the *Hdh*^*140Q/3xFlag7Q*^ striatum. 

In addition to the gliosis observed in HD, elevated levels of lipofuscin are detected in HD mouse models, and in the HD brain [43,45,46]. Lipofuscin is an autofluorescent aging pigment that accumulates over time in neurons and other postmitotic cells. It is composed primarily of cross-linked lipid, and is generated by the autophagic catabolism of membrane and organelles [47]. In HD, oxidative stress can result in the increased accumulation of perinuclear lipofuscin deposits in neurons. We assessed lipofuscin accumulation in the striatum of 24 month-old wild-type, *Hdh*^*140Q/3xFlag7Q*^, and *Hdh*^*140Q/3xFlag20Q*^, to *Hdh*^*140Q/3xFlag140Q*^ mice, and found that there was a trend towards increased lipofuscin accumulation in the striatum: wild-type<*Hdh*^*140Q/3xFlag7Q*^<*Hdh*^*140Q/3xFlag20Q*^<*Hdh*^*140Q/3xFlag140Q*^. Both *Hdh*^*140Q/3xFlag20Q*^ and *Hdh*^*140Q/3xFlag140Q*^ mice had significantly more striatal lipofuscin in comparison to wild-type mice, but there was no significant difference in the amount of striatal lipofuscin observed between wild-type and *Hdh*^*140Q/3xFlag7Q*^ mice (*P*=0.056), between *Hdh*^*140Q/3xFlag20*^ and *Hdh*^*140Q/3xFlag7Q*^ mice (*P*=0.6), or between *Hdh*^*3xFlag140Q/140Q*^ and *Hdh*^*140Q/3xFlag20Q*^ mice (*P*=0.46) (Figure 11C, D).

## Discussion

Our results are compatible with the hypothesis that the expanded polyQ stretch within mutant htt can interact in vivo with non-pathogenic lengths of polyQ in wild-type htt and potentially other cellular proteins containing a polyQ stretch. Sequestration between expanded and normal-length polyQ stretches occurs predominantly between proteolytically processed N-terminal fragments of htt, as we detect little interaction between soluble full-length 140Q-htt and 3xFlag20Q-htt. Moreover, this level of interaction is so low (<2.5% of mutant htt is associated with normal htt) that it is unlikely that sequestration is contributing to potential loss-of-function phenotypes in vivo by reducing the steady-state levels of normal htt. Our co-immunoprecipitation data also suggest that full-length wild-type mouse htt (7Q-htt) does not interact stably with itself, and supports prior work suggesting that htt is predominantly a monomeric protein in vivo [33,48,49], with the caveat that our conditions of co-immunoprecipitation select for stable interactions, and a weak or transient association between htt monomers would likely escape detection.

The number of htt inclusions detected in older mice using the aggregation-specific MW8 antibody was not altered significantly when the polyQ stretch in normal htt was increased from 7Q to 20Q, and the mouse PRR was substituted with a human PRR, despite a significant increase in the number of *Hdh*^*140Q/3xFlag20Q*^ nuclear inclusions that was observed at 6 months of age. However, the mean size of htt aggregates was significantly larger in the *Hdh*^*140Q/3xFlag20Q*^ striatum in comparison to *Hdh*^*140Q/3xFlag7Q*^ striatum at 12 and 24 months of age. A potential explanation for these observations is that an interaction between N-terminal fragments of 3xFlag20Q-htt and 140Q-htt could enhance nucleation kinetics, facilitating small htt inclusion formation in *Hdh*^*140Q/3xFlag20Q*^ mice. Larger numbers of small htt inclusions could then increase the probability that they can coalesce into larger inclusions that are recognized by the MW8 antibody in the *Hdh*^*140Q/3xFlag20Q*^ brain.

Our data obtained using formic acid to both expose the expanded polyQ epitope in tissue sections and to solubilize nuclear-associated htt aggregates, provide evidence supporting this scenario. Small cytoplasmic htt aggregates were increased at both 6 months and 24 months of age in the *Hdh*^*140Q/3xFlag20Q*^ striatum in comparison to *Hdh*^*140Q/3xFlag7Q*^ striatum, and higher levels of 3xFlag20Q-htt N-terminal fragments were detected in nuclear-associated formic acid-solubilized aggregates in comparison to 3xFlag7Q-htt fragments in age-matched controls. These data are compatible with prior in vitro observations suggesting that normal length polyQ polypeptides enhance the nucleation kinetics of an expanded polyQ polypeptide in a process that is both concentration- and polyQ length-dependent [16].

An alternative to the polyQ-dependent enhanced nucleation hypothesis that cannot be excluded by our data is that substitution of the mouse PRR with the human PRR in 3xFlag20Q-htt also contributed to the phenotypic differences between the *Hdh*^*140Q/3xFlag7Q*^ and *Hdh*^*140Q/3xFlag20Q*^ mice that we observed. Mammalian cell culture experiments with truncated N-terminal wild-type and mutant htt expression constructs have provided data suggesting that htt amino acids located C-terminal to the polyQ stretch can influence co-aggregation of mutant and wild-type htt fragments [30]. Moreover, data from both yeast and mammalian cell culture studies suggest that the htt PRR may be involved in the formation of the aggresome [28,29]. A future test of this hypothesis would require the generation of *Hdh* alleles encoding full-length 7Q-htt with the human PRR and 20Q-htt with the mouse PRR.

The htt aggregation phenotype we detect in the *Hdh*^*140Q/3xFlag20Q*^ mice was accompanied by enhanced gliosis and lipofuscin accumulation. In HD, however, it is unclear whether or not increasing the length of the CAG repeat in the normal *HTT* allele can affect pathogenesis. In a study combining data from 533 individuals from the Huntington’s Disease Modifiers of Age at Onset in Pairs of Siblings (HD-MAPS) study and 221 individuals from the Huntington Disease Center with Walls (NEHD) study, evidence was found supporting a genetic interaction between the normal and mutant *HTT* alleles – age at onset decreases when the normal CAG repeat length increases from 6–17 to 18–19 repeats, and increases when the CAG repeats range from 20–34 [50]. This effect on age-at-onset from the normal allele is small, however (1–2 year difference in age-at-onset), and may reflect the difficulty associated with determining a precise time for symptomatic onset. In a more recent study involving 921 subjects, increasing the size of the normal htt polyQ stretch correlated with a lower age-at-onset and increased clinical severity if the mutant htt polyQ stretch length was between 36Q and 44Q, while the opposite effect was observed if the mutant Htt polyQ stretch was >44Q [51]. However, two more recent studies could find no evidence supporting the hypothesis that the size of the polyQ stretch in the normal *HTT* allele influences age-at-onset [52,53]. Our data suggest that some neuropathological phenotypes are enhanced by expressing 3xFlag20Q-htt in the CAG140 mouse model. However, additional studies will be needed to determine if the age-at-onset, severity, and progression of all knock-in HD mouse model phenotypes are similarly affected. In addition, we will need to examine if substitution of the mouse PRR with the human PRR is also contributing to the differences observed between the *Hdh*^*140Q/3xFlag20Q*^ and *Hdh*^*140Q/3xFlag7Q*^ mice.

The generation of *Hdh* knock-in alleles expressing N-terminal epitope-tagged 7Q-, 20Q-, and 140Q-htt provide additional tools for understanding normal and mutant htt function in the mouse. However, we caution that the incorporation of the N-terminal epitope tag may perturb htt function. In yeast, a Flag epitope tag at the N- or C-terminus of constructs expressing truncated N-terminal fragments of mutant htt enhanced cytotoxicity [54]. Similarly, our 3xFlag or HA tags may influence the structure and function of both normal and mutant htt in our knock-in mouse models. Nevertheless, we obtained the predicted numbers of *Hdh*^*3xFlag7Q/3xFlag7Q*^ homozygotes, and *Hdh*^*3xFlag7Q/-*^ hemizygotes, suggesting that normal htt’s essential developmental functions were not affected significantly by the addition of the 3xFlag epitope tag. We also were able to obtain *Hdh*^*3xFlag140Q/3xFlag140Q*^ homozygotes from heterozygous intercrosses, but future studies comparing both the behavior and neuropathology in *Hdh*^*140Q/140Q*^ and *Hdh*^*3xFlag140Q/3xFlag140Q*^ mice are needed to determine to what extent an N-terminal tag on mutant htt could affect HD mouse model phenotypes.

## Conclusion

The generation of new *Hdh* knock-in alleles expressing N-terminal epitope-tagged htt with various polyQ lengths has allowed us to begin to explore the interaction between normal and mutant htt, and to investigate the consequence of expressing a normal htt with a 20Q stretch and human PRR in the CAG140 knock-in model for HD. Substitution of the normal mouse exon 1 sequence in the wild-type *Hdh* allele with normal human sequence can exacerbate some phenotypes in a heterozygous CAG140 knock-in mouse model for HD, suggesting that the length of the normal polyQ stretch and/or the presence of a human PRR in normal huntingtin can modulate HD mouse model pathogenesis. We hope that these new *Hdh* knock-in alleles can be of use to the research community.

## Methods

All experiments with mice were carried out in accordance with the ethical guidelines described in the “Guide for the Care and Use of Laboratory Animals”, Institute of Laboratory Animal Resources, National Research Council, 1996 edition. All procedures were reviewed and approved by the Animal Care and Use Committee of the University of Virginia.

### Generation of mice

Partially complementary oligonucleotides containing sequence encoding an HA or 3xFlag N-terminal epitope tag, an *AlwN*I restriction site at the 5´end, and an *Xmn*I restriction site at the 3´end were annealed, repaired with the Klenow fragment of DNA polymerase I, and then digested with *AlwN*I and *Xmn*I restriction enzymes to generate a synthetic DNA fragment that was used to replace an endogenouse *Hdh* exon 1 *AlwN*I–*Xmn*I fragment encoding the N-terminal amino acids of htt. Oligonucleotides used were, HA-f: 5´-GTCTTCAGGGTCTGTCCCATCGGGCAGGAAGCCGTCATGTACCCATACGACGTCCCAGACTACGCT-3´, HA-r: 5´-CGACTCGAAAGCCTTCATCAGCTTTTCCAGGGTTGCAGCGTAGTCTGGGACGTCGTATGGGTA-3´, Flag3x-f: 5´-GTCTTCAGGGTCTGTCCCATCGGGCAGGAAGCCGTCATGGACTACAAAGACCATGAGGGTGATTATAAAGATCATGACATCGACTACAAGGACGACGATGACAAG-3´, Flag3x-r: 5´-CGACTCGAAAGCCTTCATCAGCTTTTCCAGGGTTGCCTTGTCATCGTCGTCCTTGTAGTC-3´. For assembly of the *Hdh*^*3xFlag20Q*^ gene targeting vector, An *Xmn*I–*Kpn*I fragment containing human wild-type *HTT* sequence was obtained by PCR from genomic DNA according to the method described in [32]. This fragment contains the human *Xmn*I restriction site, a normal 20Q stretch, the human proline-rich region (PRR), human sequence extending to the end of exon 1, a 100 bp intron 1 deletion near the 5´-splice site of intron 1, and a *Kpn*I restriction site. For assembly of the *Hdh*^*3xFlag140Q*^ gene targeting vector, the 3xFlag epitope tag *AlwN*I–*Xmn*I restriction fragment was used to replace the corresponding *AlwN*I–*Xmn*I fragment in our CAG140 targeting vector. Thus, this vector also contains both the human PRR and intron 1 deletion. In contrast, insertion of the epitope tag sequences was the only modification made to the *Hdh*^*HA7Q*^ and *Hdh*^*3xFlag7Q*^ gene targeting vectors. Following electroporation of the targeting vectors into the W9.5 ES cell line, targeted clones were identified by Southern blot analysis as described [32]. Southern positive clones were then screened for expression of epitope-tagged full-length htt using anti-HA (HA.11, Covance) and anti-Flag (FLAG M2, Sigma) antibodies. Mice were generated from the targeted ES cell clones using standard procedures, and germline transmission was obtained from at least two independently-targeted ES cell clones for each construct. All lines were backcrossed with C57BL/6J mice for at least six generations.

### Genotyping

For genotyping the *Hdh*^*HA7Q*^ and *Hdh*^*3xFlag7Q*^ alleles, HDepi-1: 5´-GCGTAGTGCCAGTAGGCTCCAAG-3´and HDepi-2: 5´-CTGAAACGACTTGAGCGACTCGAAAG-3 amplify a portion of the *Hdh* sequence flanking the epitope tag insertion site between mouse htt amino acids 1 and 2. A wild-type *Hdh* allele will generate a 112 bp PCR product, while the *Hdh*^*3xFlag7Q*^ allele will generate a 178 bp product due to the insertion of the 22 amino acid 3xFlag epitope tag. The *Hdh*^*HA7Q*^ allele generates a 139 bp PCR product due to the insertion of the nine amino acid HA epitope tag. For genotyping the *Hdh*^*3xFlag20Q*^ and *Hdh*^*3xFlag140Q*^ alleles, HDepi-1 and HuHDepi-2: 5´-GAAGGACTTGAGGGACTCGAAGG-3´are used. HuHDepi-2 is homologous to the human sequence located 3´of the *Xmn*I restriction within exon 1 of both the *Hdh*^*3xFlag20Q*^ and *Hdh*^*3xFlag140Q*^ alleles. Both the *Hdh*^*3xFlag20Q*^ and *Hdh*^*3xFlag140Q*^ alleles will generate a 176 bp PCR product with this primer pair, while no product will be generated from the wild-type *Hdh* allele. Alternatively, the *Hdh*^*3xFlag20Q*^ and *Hdh*^*3xFlag140Q*^ alleles can be genotyped by using 140for: 5´-CTGCACCGACCGTGAGTCC-3´ and 140rev: 5´-GAAGGCACTGGAGTCGTGAC-3´. These primers flank the 100 bp intron 1 deletion that is present in the *Hdh*^*3xFlag20Q*^, *Hdh*^*3xFlag140Q*^, and *Hdh*^*140Q*^ alleles, and will generate a 150 bp PCR product, while the wild-type *Hdh*, *Hdh*^*3xFlag7Q*^, and *Hdh*^*HA7Q*^ alleles lacking the intron 1 deletion generate a 235 bp product. The CAG repeat lengths in the *Hdh*^*3xFlag20Q*^ (23Q), *Hdh*^*3xFlag140Q*^ (136Q) alleles were determined by PCR sequencing (Laragen, Inc.) of genomic DNA isolated from tail biopsies.

### Co-immunoprecipitation

Whole brain tissue was dounce homogenized in extraction buffer {50 mM Tris–HCl pH 8.8, 100 mM NaCl, 5 mM MgCl_2_, 1 mM EDTA pH 8.0, 0.5% (v/v) NP40, protease inhibitor cocktail (Thermo Scientific), with 5 mM NaF and 1 mM sodium vanadate} on ice, and then centrifuged at 16,100×g for 10´at 4°C to obtain a crude cytoplasmic supernatant fraction. Co-immunoprecipitation was performed by incubating protein extracts with either FLAG M2 antibody-agarose beads or by incubating antibody (FLAG M2 or 1C2) with the extract, followed by capture of the antigen-antibody complex with Protein G-agarose beads. In the first method, 0.5–1.0 mg of the protein lysate was incubated by rotation o/n at 4°C with 12–20 μl FLAG M2 antibody-agarose beads (Sigma) that were pre-washed three times with 0.5 ml extraction buffer according to the manufacturer’s instructions. Following incubation of the antibody-agarose beads with the extract, the beads were pelleted by centrifugation at 8,200×g for 30 sec at 4°C, and the supernatant was carefully removed and saved as the antibody non-bound fraction (NB). The beads were then washed three times with 0.5 ml ice-cold extraction buffer. Antibody-bound protein was eluted with two sequential incubations in 30 μl 0.4 mg/ml 3xFlag peptide (Sigma) dissolved in extraction buffer. Both elution fractions were then combined as the antibody-bound (B) fraction. NB- and B-samples were fractionated on 5% SDS-PAGE and then transferred electrophoretically o/n at 30V in 25 mM Tris base, 192 mM glycine, 10% methanol, 0.025% SDS to 0.45 μm PVDF membranes (Millipore). In the second method, 500 μg cytoplasmic extract was incubated o/n at 4°C with 5–6 μg FLAG M2 or 1C2 antibody. 20 μl Protein-G agarose beads (Upstate Biotechnology; washed once in extraction buffer, blocked for 1 h at 4°C in 1% blocking powder {Boehringer} dissolved in TBS, and then washed 3X in extraction buffer prior to resuspending in the original volume of the same buffer), was then added to the mixture of protein extract and antibody. This mixture was then rotated for 3 hr at 4°C to bind the antibody-antigen complexes to the beads. The beads were then pelleted by centrifugation, the supernatant was saved as the NB-fraction, and then the beads were washed as described previously for the anti-FLAG M2-agarose beads. The B-fraction was eluted from the beads by adding 50 μl 3× SDS-PAGE sample loading buffer to the beads, and incubating for 5 min at 99°C in a heating block. The beads were then pelleted, and the supernatant transferred to a fresh tube. 5% of the NB-fraction and 50% of the B-fraction were analyzed by SDS-PAGE and western blotting as described above. Western blot membranes were blocked in 5% milk in TBS-0.05% Tween 20 (TBST) prior to incubation o/n at 4°C with either FLAG M2, (Sigma), HA.11 (Covance), the expanded polyQ-specific monoclonal antibody 1C2 (MAB1574, Millipore) or MAB2166 (Millipore). Membranes were washed five times with TBST before incubation with secondary goat anti-mouse IgG-HRP conjugated antibodies (Pierce) for 1 h at room temperature. Following an additional five washes, the membranes were incubated with West-Dura chemiluminescence substrate (Pierce) and then exposed to film. For quantification of the blots, exposures within the linear range of the film were scanned, and then analyzed by Image J software (http://imagej.nih.gov/ij/download.html). The percentage of 140Q-htt co-immunoprecipitating with 3xFlag20Q-htt was determined by dividing the pixel intensity of the B-fraction 140Q-htt signal by the NB-fraction signal, and then adjusting for the fraction of each sample loaded, and also for the efficiency of 3xFlag20Q-htt immunoprecipitation as determined from the 2166 western blots (e.g. ~20% of 3xFlag20Q-htt was immunoprecipitated in the experiment shown in Figure 5C, and the % of 140Q-htt associating with 3xFlag20Q-htt would then be corrected by a factor of 100/20).

### Western blot analysis of formic acid-solubilized htt aggregates

Brains were cut in half (sagittally), and each half brain was dounce-homogenized in 2 ml extraction buffer on ice, before centrifugation at 800×g for 15´ at 4°C to obtain a crude nuclear pellet fraction. Formic acid-solubilized nuclear htt aggregates were obtained following the procedures of [39] and [40] with modifications. The crude nuclear fraction was first resuspended in extraction buffer, and then boiled 15 min in the presence of SDS prior to sonication with a Fisher Scientific 550 probe sonicator (power level 5 for 20 sec with pulses of 0.6 sec on/0.4 sec off). Following sonication, the sample was centrifuged at 16,100xg for 15´at RT, and the SDS-insoluble pellet was resuspended in formic acid and incubated for 1 h at 37°C at 350 rpm. The sample was then dried in a speed-vac and resuspended in 1M Tris base to neutralize residual formic acid. An equal volume of 2X SDS-PAGE loading buffer was added, and then one third of the sample was incubated 5 min at 97°C prior to fractionation on 4%–15% gradient SDS-PAGE (BioRad) and blotting onto 0.45 μm PVDF membrane. Blots were probed with Flag (FLAG M2, Sigma) and expanded polyQ-specific (1C2, Millipore) antibodies, and imaged using a ChemiDoc XRS+ (BioRad) with West-Dura chemiluminescence reagents (Pierce).

### Filter trap assay for htt aggregation

Whole brains were dounce homogenized in extraction buffer and centrifuged at 800×g for 15´at 4°C to obtain a crude nuclear pellet fraction. Aliquots of this fraction were treated for 60 minutes on ice with 0.1 mg/ml DNAse I, SDS was then added to 2% final concentration, and incubated 5 minutes at 99°C prior to spotting onto a cellulose acetate/PVDF membrane sandwich. SDS-resistant htt inclusions are trapped on the cellulose acetate membrane while soluble 3xFlag7Q-htt and 140Q-htt species are retained on the underlying PVDF membrane. Htt inclusions were detected by western blotting with the 1C2 and MAB5374 antibodies, while sequestered 3xFlag7Q-htt was detected with the FLAG M2 antibody.

### Immunohistochemistry

Dissected brains were flash-frozen in isopentane on dry ice, and then serially sectioned at 14 μm on a cryostat (Bright Instrument Co.). Sections were either used immediately or stored at −80°C until use. Sections were washed briefly in PBS, fixed for 10 min in 4% paraformaldeyde in 0.1M phosphate buffer pH 7.4, and washed in PBS three times before blocking with a 1:100 dilution of monovalent Fab fragments (donkey anti-mouse IgG (H+L), Jackson ImmunoResearch) in blocking buffer (5% donkey serum, 0.1% Triton X100 in PBS) for 1 h at RT, followed by three washes in PBS, and then incubated o/n at 4°C with primary antibody diluted in blocking buffer. Primary antibodies used were mouse monoclonal MW8 (1:70, Developmental Studies Hybridoma Bank), mouse monoclonal MAB5375 (1:100, mEM48, Millipore), mouse monoclonal FLAG M2 (1:100, Sigma), mouse monoclonal HA.11 (1:100, Covance), and rabbit anti-GFAP (1:1000, AB5804, Millipore). Following incubation with the primary antibody, sections were washed three times in PBS, and then incubated with secondary antibody (Cy3- or FITC-conjugated donkey anti-mouse or donkey anti-rabbit-IgG, Jackson ImmunoResearch) together with the fluorescent DNA stain To-Pro-3 iodide (Invitrogen) in blocking buffer for 1 h at RT. Sections were then washed with PBS before treating with Autofluorescence Eliminator Reagent (Millipore) following the manufacturer’s instructions. Sections were then mounted with Vectashield (Vector Laboratory) and examined using either an Olympus BX51 microscope equipped with a MagnaFire CCD camera or a Nikon C1 confocal system.

### Immunohistochemical detection of htt aggregates following formic acid treatment

The method described in [41] was adapted for fresh frozen sections. Sections were washed briefly in PBS, and fixed in 4% paraformaldehyde in 0.1M PB for 15 min at RT. Sections were then washed twice in PBS, once with water (10 min each), and then treated with formic acid (Sigma) three times for 10 min each. The sections were rinsed three times in water and washed twice for 10 min each in PBS, followed by 30 min incubation in 1% sodium borohydride (w/v) in PBS, and then washed three times for 10 min each in PBS prior to immunohistochemical staining using the monoclonal 1C2 antibody (1:100, Millipore).

### Quantification of htt aggregates using the MW8 antibody

Tissue sections were imaged with a 60X objective using a Nikon C1 confocal microscope, and the number of MW8^+^ huntingtin aggregates was counted blind to genotype from sagittal sections through the striatum (8 images from sections separated by 280 μm through the striatum, n=4 mice of each genotype) using ImagePro 4.5 software (Media Cybernetics). Neuropil and nuclear inclusions were counted separately. To determine the mean size of inclusions, the total pixel area was divided by the number of inclusions in each imaging field.

*Lipofuscin analysis.* Imaging and quantification of lipofuscin was performed as described in [46].

### Statistical analyses

χ^2^ analyses of the genotypes were performed using SigmaStat (Systat software). Student’s *t*-test was used to evaluate significance in the quantification of aggregate numbers, aggregate mean size, and co-localization of MW8 and 3xFlag epitopes in htt aggregates.

## Competing interests

The authors declare that they have no competing interests.

## Authors’ contributions

SZ characterized the phenotypes of the epitope-tagged *Hdh* knock-in mice, NG participated in the immunohistochemical analyses and generated the epitope-tagged *Hdh* knock-in mice, JB generated the targeting constructs and the targeted ES cell clones, J-PL carried out the formic acid western analyses and participated in drafting the manuscript, and SOZ conceived of the study, participated in its design and coordination, performed some of the co-immunoprecipitation experiments, and helped to draft the manuscript. All authors read and approved the final manuscript.
